# The Geographical Distribution and Burden of Trachoma in Africa

**DOI:** 10.1371/journal.pntd.0002359

**Published:** 2013-08-08

**Authors:** Jennifer L. Smith, Rebecca M. Flueckiger, Pamela J. Hooper, Sarah Polack, Elizabeth A. Cromwell, Stephanie L. Palmer, Paul M. Emerson, David C. W. Mabey, Anthony W. Solomon, Danny Haddad, Simon J. Brooker

**Affiliations:** 1 London School of Hygiene and Tropical Medicine, London, United Kingdom; 2 International Trachoma Initiative, Task Force for Global Health, Atlanta, Georgia, United States of America; 3 The Carter Center, Atlanta, Georgia, United States of America; 4 Kenya Medical Research Institute-Wellcome Trust Research Programme, Nairobi, Kenya; University of California San Francisco, United States of America

## Abstract

**Background:**

There remains a lack of epidemiological data on the geographical distribution of trachoma to support global mapping and scale up of interventions for the elimination of trachoma. The Global Atlas of Trachoma (GAT) was launched in 2011 to address these needs and provide standardised, updated and accessible maps. This paper uses data included in the GAT to describe the geographical distribution and burden of trachoma in Africa.

**Methods:**

Data assembly used structured searches of published and unpublished literature to identify cross-sectional epidemiological data on the burden of trachoma since 1980. Survey data were abstracted into a standardised database and mapped using geographical information systems (GIS) software. The characteristics of all surveys were summarized by country according to data source, time period, and survey methodology. Estimates of the current population at risk were calculated for each country and stratified by endemicity class.

**Results:**

At the time of writing, 1342 records are included in the database representing surveys conducted between 1985 and 2012. These data were provided by direct contact with national control programmes and academic researchers (67%), peer-reviewed publications (17%) and unpublished reports or theses (16%). Prevalence data on active trachoma are available in 29 of the 33 countries in Africa classified as endemic for trachoma, and 1095 (20.6%) districts have representative data collected through population-based prevalence surveys. The highest prevalence of active trachoma and trichiasis remains in the Sahel area of West Africa and Savannah areas of East and Central Africa and an estimated 129.4 million people live in areas of Africa confirmed to be trachoma endemic.

**Conclusion:**

The Global Atlas of Trachoma provides the most contemporary and comprehensive summary of the burden of trachoma within Africa. The GAT highlights where future mapping is required and provides an important planning tool for scale-up and surveillance of trachoma control.

## Author Summary

In order to target resources and drugs to reach trachoma elimination targets by the year 2020, data on the burden of disease are required. Using prevalence data in African countries derived from the Global Atlas of Trachoma (GAT), the distribution of trachoma continues to be focused in East and West Sub-Saharan Africa, North Africa and a few endemic countries in Central Sub-Saharan Africa. Currently, 129.4 million people are estimated to live in areas that are confirmed to be trachoma endemic and 98 million are known to require access to the SAFE strategy. The maps and information presented in this work highlight the GAT as important open-access planning and advocacy tool for efforts to finalize trachoma mapping and assist national programmes in planning interventions.

## Introduction

The last decade has witnessed tremendous progress towards the global elimination of trachoma, the leading infectious cause of blindness worldwide. Since the establishment in 1998 of the Global Elimination of Trachoma by 2020 (GET2020) initiative, an increasing number of endemic countries have implemented national programmes incorporating the SAFE strategy of Surgery to correct trichiasis, Antibiotic to clear *Chlamydia trachomatis* infection, Facial cleanliness and Environmental improvement to reduce transmission. Morocco was one of the first countries in Africa to implement SAFE at the national level and achieved its Ultimate Intervention Goals (UIG) in 2006 [1]. Several other African countries, including The Gambia and Ghana, are in the post-endemic surveillance stage [2]. Supporting these country efforts there has in the last 24 months been increased political commitment by the global health community to the elimination of trachoma, as part of coordinated neglected tropical disease (NTD) control programmes, with a commensurate increase in funding. To fully realise the goals of GET2020, it will be necessary to scale up to a full SAFE programme in all endemic districts in every country by 2016–2018 in order to allow sufficient time for programme impact. Yet for some countries, especially those in Africa, there still remains a lack of epidemiological data on the geographical distribution of trachoma and efforts are required to first complete the global trachoma map, then to keep it updated as interventions begin to take effect. This will help inform where and when to start and stop trachoma control efforts.

To respond to this need, the Global Atlas of Trachoma (www.trachomaatlas.org) was launched in early 2011 as a collaborative venture between the London School of Hygiene and Tropical Medicine (LSHTM), The Carter Center, and the International Trachoma Initiative (ITI). It provides regularly updated, open-access district-level prevalence maps of the current distribution of trachoma [3]. These maps and the underlying database provide a planning tool that can help to define the known geographic distribution of trachoma at sub-national levels and identify gaps in survey data where further mapping is required. The aim of this paper is to describe the geographical distribution of trachoma in Africa using existing data from the Global Atlas of Trachoma and estimate the burden of disease in Africa. Specifically, we will describe the methods of data assembly and mapping and use these data to define the current geographical distribution, calculate the population at risk of TF and TT, and estimate numbers requiring treatment. We also assess the remaining effort required to finalise the mapping of trachoma on the continent.

## Methods

### Overview

The Global Atlas of Trachoma has adopted an identification and data assembly strategy similar to other mapping initiatives, including those for malaria [4], helminth infections [5]–[7] and human African trypanosomiasis [8]. In brief, epidemiological data on the burden of trachoma are identified through structured searches of published and unpublished literature, with a number of inclusion rules applied to identified information. Data are then abstracted into a standardised database and mapped using geographical information systems (GIS) software.

### Estimating the burden of trachoma

The burden of trachoma in a given community is typically measured by the prevalence of clinical signs of disease. This diagnosis is based on ocular examination, usually using the 1987 WHO simplified grading system, to identify the presence of key clinical signs [9]: trachomatous inflammation–follicular (TF) in children aged 1–9 years and trachomatous trichiasis (TT) in adults aged over 14 years. Although the presence of TF does not always correspond to infection with *C. trachomatis* infection [10], these measures are easily collected in the field and used to guide the planning and implementation of the SAFE strategy at the district (second administrative) level [11]. These indicators are also used to define GET2020 UIGs, which are less than one case of TT unknown to the health system per 1000 total population and <5% TF in children aged 1–9 years, at the district, sub-district or community level [12].

Surveys assessing the burden of trachoma use one of four methodologies: population based prevalence surveys (PBPS); acceptance sampling trachoma rapid assessment (ASTRA); “Integrated Threshold Mapping” (ITM); or trachoma rapid assessment (TRA) [13]. PBPS are the preferred method since they provide a representative measure of the prevalence of trachoma within a population. The most common PBPS strategy is cluster randomized sampling (CRS) which uses a representative, two-stage sampling methodology to provide a “gold” standard prevalence estimate at the district level, used for targeting SAFE interventions including mass drug administration (MDA) according to treatment thresholds. General guidelines recommend sampling 20 clusters per district, although this varies in practice, and the precision of prevalence estimates is rarely reported [14]. TRA was developed as a rapid and inexpensive method using convenience sampling to rank communities in terms of priority for control programmes [15]. TRAs are “optimally biased” to find trachoma where it is endemic, and do not provide a reliable estimate of trachoma prevalence; a negative TRA probably reliably identifies the absence of trachoma. ASTRA is a form of lot quality assurance sampling and can reliably classify communities in relation to a threshold value [16], but has in practice been rarely used as it requires modification to derive overall population estimates of trachoma prevalence. More recently, ITM has been developed; it employs convenience sampling of school children, pre-school children and women of child-bearing age to determine whether the prevalence of trachoma, as well as prevalences of other NTDs, exceed some designated threshold, with initial piloting having been conducted in Mali and Senegal [17] and further use in Togo and Zambia.

### Identification of survey data

To assemble a global database of trachoma risk, survey data were identified through a combination of (i) searches of electronic bibliographic databases; (ii) review of programmatic data submitted to the International Trachoma Initiative (ITI); (iii) manual searches of local archives and WHO GET2020 documents; and (iv) direct contact with programme managers and researchers. These searches, conducted in 2010 and annually thereafter, build on an earlier effort in 2003 as part of a collaboration between the International Centre for Eye Health at the London School of Hygiene and Tropical Medicine (LSHTM) and the Programme for the Prevention of Blindness and Deafness at the WHO, to develop a first global atlas of trachoma [18]. The online bibliographic databases PubMed and Embase were searched to identify relevant studies, using the Medical Subject Headings trachoma, trichiasis, and *Chlamydia trachomatis*. These searches were restricted to surveys conducted after 1980 for trichiasis and 1988 for active trachoma. The latter restriction was applied because 1987 is when the new simplified grading system for trachoma was introduced [9]. Authors were contacted if additional information was required on survey design or indicators collected. Countries for which no up-to-date information was available from the literature, GET 2020 country data forms, or submitted to ITI, were contacted on an individual basis for local knowledge and clarification. As a whole, these data are unpublished and use the standardised survey methodologies recommended by WHO. Work initially focused on the 53 countries classed in 2004 as trachoma endemic by The World Health Organization, 36 of which are in Africa [19]. There is currently no reliable data indicating the status of trachoma in Libya, Namibia or Zimbabwe. These countries were therefore not included in the analysis. The aim was also to collect the most contemporary data possible in order to inform current control efforts. Literature searches are conducted annually (most recently in April, 2012), and additional data submitted directly to ITI by national trachoma program managers are routinely used to update the database and resulting prevalence maps. Data available as of September, 2012, were used in the preparation of this manuscript.

### Data selection and entry

The title and abstract of each source of information were reviewed and evaluated against a number of pre-defined inclusion and exclusion criteria: only cross-sectional population based prevalence surveys were included as measures of trachoma prevalence; TRAs were only used to indicate the presence or absence of trachoma where no prevalence data were available. Data were excluded if based on hospital or clinic surveys, or surveys among sub-populations such as among refugee populations. Where multiple surveys were available from the same district but surveyed at different times, they were included as separate entries and coded as “current” or “historical” in order to ensure that only the most recent data are used to estimate the current burden of disease. Estimates of disease prevalence were typically available at the district level as this is the administrative unit at which control is implemented. Where estimates were representative of point locations or the result of a non-random selection of communities within a district, data were only used to provide information on the presence of trachoma. Abstracted data included details on the source of the data, location of survey (including geographical co-ordinates for cluster data when available), survey year, characteristics of the surveyed population, survey methodology, the numbers of children aged 1–9 years and adults aged over 14 years examined, the number of children graded positive for TF and the number of adults graded positive for TT. Any variation in clinical indicator or age group was also recorded in the database. A unique identifier linked each record in a bibliographic database to the survey data and to an electronic copy of the source when this could be obtained.

### Mapping

All data were entered into a standardized Microsoft Access 2007 geodatabase (Microsoft Corporation, Redmond, WA, USA), which is linked to a geographic information system (GIS). Data can be queried to produce custom tables, thus allowing simple and rapid generation of country and regional maps using Arc GIS 10.1 (ESRI, Redlands, CA, USA). Data were assigned wherever possible to the second administrative level (e.g., district level), which has direct relevance to implementation of trachoma control. However, data from some older surveys and hyperendemic areas are available at the first administrative level (e.g., province, region), and data were assigned accordingly. The most recent data are displayed on the main maps. Where historical data are also available they are displayed on separate maps online.

Prevalence data were banded into categories corresponding to current intervention guidelines for TF and TT (Table 1). TRA data were categorized into three bands for active trachoma (No active trachoma found, <10% and ≥10% of children aged 1–9 years examined found positive) and two bands for trichiasis corresponding to UIG targets (<0.1% and ≥0.1% of the total population examined found positive). Geographical boundaries used for mapping were derived from: (i) the United Nations Second Administrative Level Boundaries data set project (http://www.unsalb.org/), (ii) Global Administrative Areas (http://www.gadm.org/), and (iii) shapefiles created specifically for this project from maps provided by programme managers. Updated district-level maps were launched in 2011 on an open-access website (www.trachomaatlas.org).

**Table 1:** Endemicity classes for implementation of SAFE based on trachomatous inflammation–follicular (TF) and trichiasis (TT).

TF Prevalence band	Classification	Implementation
<5%	Non-endemic	No need for implementation of AFE
≥5% and <10%	Hypo-endemic	Mapping, F and E can be applied, focal A
≥10% and <30%	Meso-endemic	AFE at district level (≥3 years then review)
≥30%	Hyper-endemic	AFE at district level (≥5 years then review)

TT Prevalence band	Classification
<0.1%	UIG achieved
≥0.1%	UIG not yet achieved

### Analysis

The characteristics of all surveys that met the inclusion criteria were summarized by country according to data source, time period, and survey methodology. Districts and regions were categorized as suspected endemic or assigned to a prevalence category using the most current PBPS data representative at this level. Districts were classified as ‘suspected endemic’ or ‘suspected non-endemic’ based on information from TRA surveys, point locations, reported cases or anecdotal information from national programs. Surveys which only collected data on one clinical sign were also used to inform this classification (i.e. a district known to be endemic for TF was classified as ‘suspected endemic’ for TT where no other data were available). Note that, in some cases, identified districts may not include all endemic or non-endemic areas within a country, but their classification does reflect available evidence supporting the presence of trachoma. A total of 24 district-level surveys of TT were conducted in populations aged 40 or 50 years and over. Based on a review of age-stratified TT prevalence ratios from published and unpublished data, a conversion factor of 0.54 was applied to estimate the corresponding prevalence in adults aged ≥15 years. Survey data at the region and district level were presented separately in this analysis, with district defined here as the unit of implementation typically used for SAFE control activities. While this is usually the second administrative unit within a country, in some cases these are distinct health districts (Cameroon and Burkina Faso), third administrative areas (Ethiopia) or first administrative areas (Chad, Guinea-Bissau and CAR). This was based on the aim to present data most relevant to current guidelines relating to the implementation of SAFE control strategies.

Estimates of the current population at risk were calculated for each country using district-level population estimates and summarised by endemicity class (Table 1). Population figures were derived from the Afripop project, which provided a continental 1 km gridded population map produced using projected population census data and settlement extents (www.afripop.org) [20]. This map was overlaid with district classification to allow summation and mapping of the population in each category of risk.

## Results

### Survey database and geographical coverage

A total of 167 unique surveys with data on either active trachoma or trichiasis met GAT inclusion criteria. These included data from CRS (152), ASTRA (1), TRA (9), ITM (2) and surveys at single sites (3) from 31 of the 33 countries in Africa classified as endemic. Prevalence data on active trachoma were available in 29 countries and data on TT in 25. In total, there are 1342 records included in the database representing surveys conducted between 1985 and 2012, 1253 of which provide implementation unit-level estimates of prevalence (usually district-level) and an additional 79 records that provide region–level estimates. The remaining 10 records were site-specific surveys or those of unclear methodology, which were used to provide information on the presence or absence of trachoma at the district level.

**Figure 1:**
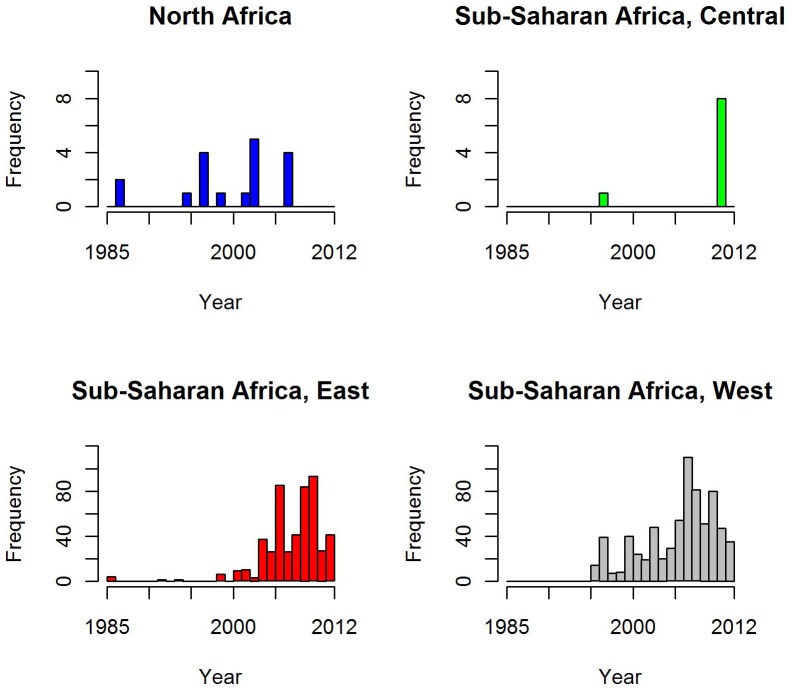
The number of prevalence surveys identified by year and region in Africa, 1985–2012. The graphs show a shift in survey activities from North Africa to other endemic areas, with a recent increase in the number of surveys conducted since 2005 in sub-Saharan Africa.

The primary source of included survey data was direct contact with national control programmes and academic researchers (67%), followed by peer-reviewed publications (17%) and unpublished reports or theses (16%). These sources of data were found to vary considerably by country with a good deal of overlap between sources in countries with established control programmes (Table 2). The number of surveys available has consistently increased over the last two decades, as highlighted in Figure 1 which presents the total number of PBPS surveys conducted by year for each region of Africa. Surveys in north Africa and the Middle East were conducted earlier than other regions, mainly reflecting active control programmes in Morocco and some earlier surveys in Egypt. While west Africa has some historical surveys, recent survey activities are increasingly focused in this region and in east Africa.

**Table 2:** Total number of district-level population based prevalence surveys (PBPS),trachoma rapid assessments (TRA) and site-specific surveys in database from countries in Africa, summarised by source of data.

					Primary source n (%)		
Country	Total number surveys	Number TRA	Number PBPS	Number Other^a^	Direct contact^b^	Published papers	Reports
Algeria	1	0	1	0	0	1 (100)	
Benin	0	0	0	0	0	0	0
Botswana	0	0	0	0	0	0	0
Burkina Faso	108	0	108	0	101 (94)	0	7 (6)
Burundi	23	0	23	0	10 (43)	13 (57)	0
Cameroon	41	0	41	0	40 (98)	1 (2)	0
CAR	10	1	9	0	2 (20)	1 (10)	7 (70)
Chad	8	0	8	0	0	5 (63)	3 (38)
Cote d' Ivoire	6	0	6	0	0	0	6 (100)
Djibouti	4	0	0	4	0	4 (100)	0
Egypt	5	1	2	2	0	5 (100)	0
Eritrea	36	0	36	0	36 (100)	0	0
Ethiopia	138	0	138	0	90 (65)	21 (15)	27 (20)
Ghana	62	1	61	0	0	61 (98)	1 (2)
Guinea	20	5	15	0	0	0	20 (100)
Guinea Bissau	9	0	9	0	9 (100)	0	0
Kenya	32	2	30	0	29 (91)	0	3 (9)
Malawi	5	0	5	0	1 (20)	4 (80)	0
Mali	62	1	61	0	29 (47)	24 (39)	9 (15)
Mauritania	64	2	62	0	64 (100)	0	0
Morocco	13	0	13	0	13 (100)	0	0
Mozambique	6	0	6	0	3 (50)	0	3 (50)
Niger	53	6	47	0	53 (100)	0	0
Nigeria	282	87	195	0	157 (56)	19 (7)	106 (38)
Senegal	11	0	10	1	11 (100)	0	0
Somalia	0	0	0	0	0	0	0
South Sudan	43	13	30	0	24 (56)	17 (40)	2 (5)
Sudan	93	0	92	1	92 (99)	1 (1)	0
Tanzania	66	0	66	0	58 (88)	8 (12)	0
The Gambia	46	0	46	0	30 (65)	16 (35)	0
Togo	31	0	28	3	3 (10)	28 (90)	0
Uganda	38	0	38	0	38 (100)	0	0
Zambia	26	0	26	0	8 (31)	0	18 (69)
**Total**	**1342**	**119**	**1212**	**11**	**901 (67.1)**	**229 (17.1)**	**212 (15.8)**

a Site specific surveys or those in which the sampling methodology was unclear and have been used to provide evidence of suspected endemicity where no district level PBPS or TRA were available;

b Direct contact includes contact with National Control Programmes, NGOs and academic researchers.

**Figure 2:**
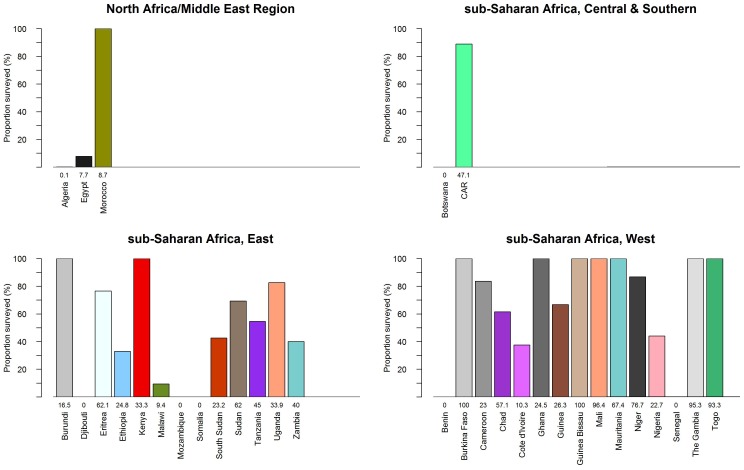
Proportion of districts surveyed by population based prevalence surveys between 1985 and 2012 in Africa. Bar plots exclude non-endemic areas from the denominator where information on suspected endemicity is available for the entire country, while numbers indicate the proportion of all districts surveyed. The graph highlights progress in mapping many endemic countries in east and west sub-Saharan Africa and the need for additional surveys in many countries in Africa.

The 33 African countries endemic for trachoma consist of 5308 districts. Of these, 1095 (20.6%) districts had representative TF data collected through PBPSs, 1024 (19.3$) with PBPS prevalence estimates for TT (Tables 3 & 4), and data from TRA surveys for an additional 101 districts. While the majority of data collected at the first administrative level are outdated and have been replaced by more recent second administrative level surveys, Tables 5 and 6 present current data on TF and TT available at this level. Only 5 first administrative level units have trachoma prevalence data that are being used programmatically. At the time of writing, 38% of the trachoma endemic countries in Africa have more than 50% of their districts mapped by PBPS and this number is even higher when excluding districts presumed to be non-endemic from the denominator, as illustrated in Figure 2. These data reflect a rise in the number of large-scale national or regional surveys taking place in recent years (e.g. in Republic of Sudan and South Sudan) as well as conduct of pre-and post-implementation surveys in the context of large-scale control programmes in several countries. Since 2007, surveys have been conducted in a number of countries that previously had no data, including Burundi, Cameroon, Central African Republic, Cote d'Ivoire, Eritrea, Rwanda, Uganda and Zambia. While a number of other countries have seen a rise in survey activities during this period (e.g. Ethiopia, Guinea Bissau, Nigeria, Republic of Sudan, South Sudan, Tanzania, Togo and Zambia), prevalence estimates are still lacking in Algeria, Chad and Djibouti and no data are currently available for Benin, Botswana, or Somalia (Tables 3 & 4).

**Table 3:** Population estimates in each endemic category of trachomatous inflammation–follicular (TF) and availability of current district level data from population based prevalence surveys (PBPS) in Africa in children aged 1–9 years.

								Prevalence of TF from PBPS^b^											
					Suspected endemic			<5%			5–9.9%			10–29.9%			>30%		
Country	Total number districts	Total pop (000s)	Total surveyed districts		Districts		Pop (000s)	Districts		Pop (000s)	Districts		Pop (000s)	Districts		Pop (000s)	Districts		Pop (000s)
			n	(%)	n	(%)^a^		n	(%)		n	(%)		n	(%)		n	(%)	
Algeria	1,592	36,507	1	(0.1)				0	(0.0)	0	0	(0.0)	0	0	(0.0)	0	1	(100)	33
Benin	77	9,307	0	(0.0)	6	(7.8)	2,192	0	(0.0)	0	0	(0.0)	0	0	(0.0)	0	0	(0.0)	0
Botswana	25	1,877	0	(0.0)	3	(12.0)	338	0	(0.0)	0	0	(0.0)	0	0	(0.0)	0	0	(0.0)	0
Burkina Faso	63	16,806	63	(100)	0	(0.0)	0	24	(38.1)	7,497	16	(25.4)	3,805	23	(36.5)	5,504	0	(0.0)	0
Burundi	139	9,681	23	(16.5)	0	(0.0)	0	11	(47.8)	2,263	8	(34.8)	1,210	4	(17.4)	965	0	(0.0)	0
Cameroon	178	20,416	41	(23.0)	8	(5.8)	948	20	(48.8)	1,157	4	(9.8)	434	15	(36.6)	1,544	2	(4.9)	72
CAR^c^	17	4,540	8	(47.1)	1	(11.1)	194	0	(0.0)	0	2	(25.0)	1,853	3	(37.5)	749	3	(37.5)	596
Chad^c^	14	12,113	8	(57.1)	5	(83.3)	4,320	0	(0.0)	0	0	(0.0)	0	5	(62.5)	3,958	3	(37.5)	3,533
Cote d' Ivoire	58	19,790	6	(10.3)				5	(83.3)	1,594	1	(16.7)	306	0	(0.0)	0	0	(0.0)	0
Djibouti	11	791	0	(0.0)	0	0.0	0	0	(0.0)	0	0	(0.0)	0	0	(0.0)	0	0	(0.0)	0
Egypt^g^	26	80,095	2	(7.7)	3	(12.5)	11,704	0	(0.0)	0	0	(0.0)	0	0	(0.0)	0	2	(100)	8,226
Eritrea	58	5,485	36	(62.1)	11	(50.0)	731	19	(52.8)	2,013	8	(22.2)	968	8	(22.2)	984	1	(2.8)	92
Ethiopia^d^	928	86,132	230^f^	(24.8)	470	(67.3)	32,586	2	(0.9)	257	4	(1.7)	562	78	(33.9)	5,678	146	(63.5)	4,069
Ghana	143	25,305	35	(24.5)	0	(0.0)	0	35	(100.0)	4,366	0	(0.0)	0	0	(0.0)	0	0	(0.0)	0
Guinea	38	10,957	10	(26.3)	5	(17.9)	785	0	(0.0)	0	0	(0.0)	0	5	(50.0)	1,577	5	(50.0)	1,029
Guinea Bissau^c^	9	1,646	9	(100)	0	(0.0)	0	0	(0.0)	0	1	(11.1)	229	7	(77.8)	1,152	1	(11.1)	213
Kenya	75	38,862	25	(33.3)	0	(0.0)	0	6	(24.0)	1,344	6	(24.0)	1,855	10	(40.0)	1,991	3	(12.0)	346
Malawi	32	14,460	3	(9.4)	5	(17.2)	2,609	0	(0.0)	0	0	(0.0)	0	3	(100)	1,290	0	(0.0)	0
Mali	55	15,864	53	(96.4)	0	(0.0)	0	32	(60.4)	9,147	11	(20.8)	3,454	10	(18.9)	1,317	0	(0.0)	0
Mauritania	46	4,260	31	(67.4)	0	(0.0)	0	20	(64.5)	1,137	8	(25.8)	299	2	(6.5)	20	1	(3.2)	764
Morocco	46^e^	31,954	4	(8.7)	0	(0.0)	0	4	(100)	1,719	0	(0.0)	0	0	(0.0)	0	0	(0.0)	0
Mozambique	132	22,467	0^f^	(0.0)	106	(80.3)	16,580	0	(0.0)	0	0	(0.0)	0	0	(0.0)	0	0	(0.0)	0
Niger	43	16,196	33	(76.7)	5	(50.0)	1,233	10	(30.3)	4,375	3	(9.1)	1,111	14	(42.4)	5,942	6	(18.2)	3,097
Nigeria	774	160,067	176	(22.7)	224	(37.5)	46,132	53	(30.1)	10,039	39	(22.2)	6,945	66	(37.5)	14,644	18	(10.2)	3,569
Senegal	44	12,034	0^f^	(0.0)				0	(0.0)	0	0	(0.0)	0	0	(0.0)	0	0	(0.0)	0
Somalia	74	8,958	0	(0.0)				0	(0.0)	0	0	(0.0)	0	0	(0.0)	0	0	(0.0)	0
South Sudan	99	9,606	23	(23.2)	31	(40.8)	3,523	3	(13.0)	194	0	(0.0)	0	1	(4.3)	150	19	(82.6)	1,931
Sudan	142	32,376	88	(62.0)	39	(72.2)	7,266	73	(83.0)	18,634	12	(13.6)	3,206	3	(3.4)	381	0	(0.0)	0
Tanzania	120	43,494	54	(45.0)	45	(68.2)	17,976	6	(11.1)	2,445	6	(11.1)	2,196	25	(46.3)	7,464	17	(31.5)	4,027
The Gambia	43	1,719	41	(95.3)	0	(0.0)	0	21	(51.2)	617	13	(31.7)	569	7	(17.5)	139	0	(0.0)	0
Togo	30	5,944	28	(93.3)	0	(0.0)	0	28	(100)	5,649	0	(0.0)	0	0	(0.0)	0	0	(0.0)	0
Uganda	112	32,415	38	(33.9)	8	(10.8)	1,899	1	(2.6)	1,753	3	(7.9)	1,289	20	(52.6)	5,450	14	(36.8)	3,848
Zambia	65	12,004	26	(40.0)	28	(71.8)	4,072	5	(19.2)	522	5	(19.2)	781	14	(53.8)	1,697	2	(7.7)	304
**Total**	**5,308**	**804,128**	**1095**	**(20.6)**	**1,003**	**23.8**	**155,086**	**378**	**(34.5)**	**76,722**	**150**	**(13.7)**	**31,072**	**323**	**(29.5)**	**62,596**	**244**	**(22.3)**	**35,749**

a Proportion of unsurveyed districts that are suspected endemic.

b Proportion of known endemic districts falling into each category of endemicity.

c Unit of implementation (health district) is defined as the first administrative level.

d Third administrative level (wereda) is the implementation unit, but some zonal data are included in this table and used to inform SAFE implementation.

e Five districts were historically endemic in Morocco.

f Regional data available in Table 5.

g Data in Egypt were collected at the governorate (regional) level, there have been no recent surveys at finer spatial scales and no alternative public health districts have been defined.

**Table 4:** District estimates in each endemic category of trichaisis (TT) and availability of current district level data from population based prevalence surveys (PBPS) in Africa in adults aged greater than 15 years.

								Surveyed by PBPS^b^			
					Suspected endemic			<0.1%		≥0.1%	
Country	Total number districts	Total population (000s)	Total surveyed districts		Districts		Pop (000s)	Districts		Districts	
			n	(%)	n	(%)^a^		n	(%)	n	(%)
Algeria	1,592	36,507	0	(0.0)	1	(0.1)	33	0	(0.0)	0	(0.0)
Benin	77	9,307	0	(0.0)	6	(7.8)	2,192	0	(0.0)	0	(0.0)
Botswana	25	1,877	0	(0.0)	3	(12.0)	338	0	(0.0)	0	(0.0)
Burkina Faso	63	16,806	63	(100)	0	(0.0)	0	6	(9.5)	57	(90.5)
Burundi	139	9,681	0	(0.0)	4	(2.9)	965	0	(0.0)	0	(0.0)
Cameroon	178	20,416	41	(23.0)	8	(5.8)	948	15	(36.6)	26	(63.4)
CAR^c^	17	4,540	9	(52.9)	1	(12.5)	194	1	(11.1)	8	(88.9)
Chad^c^	14	12,113	8	(57.1)	5	(83.3)	4,320	0	(0.0)	8	(100)
Cote d' Ivoire	58	19,790	6	(10.3)				4	(66.7)	2	(33.3)
Djibouti^g^	11	791	0	(0.0)	4	(36.4)	580	0	(0.0)	0	(0.0)
Egypt^h^	26	80,095	2	(7.7)	3	(12.5)	11,704	0	(0.0)	2	(100)
Eritrea	58	5,485	36	(62.1)	11	(50.0)	731	14	(38.9)	22	(61.1)
Ethiopia^d^	928	86,132	202	(21.8)	470	(64.7)	32,586	1	(0.5)	201	(99.5)
Ghana	143	25,305	35	(24.5)	0	(0.0)	0	15	(42.9)	20	(57.1)
Guinea	38	10,957	15	(39.5)	0	(0.0)	0	0	(0.0)	15	(100)
Guinea Bissau^c^	9	1,646	9	(100)	0	(0.0)	0	0	(0.0)	9	(100)
Kenya	75	38,862	13	(17.3)	7	(11.3)	258	0	(0.0)	13	(100)
Malawi	32	14,460	3	(9.4)	5	(17.2)	2,609	0	(0.0)	3	(100)
Mali	55	15,864	53	(96.4)	0	(0.0)	0	2	(3.8)	51	(96.2)
Mauritania	46	4,260	31	(67.4)	0	(0.0)	0	12	(38.7)	19	(61.3)
Morocco^e^	46	31,954	5	(10.9)	0	(0.0)	0	0	(0.0)	5	(100)
Mozambique	132	22,467	0	(0.0)	106	(85.3)	16,580	0	(0.0)	0	(0.0)
Niger	43	16,196	33	(76.7)	2	(20.0)	137	6	(18.2)	27	(81.8)
Nigeria	774	160,067	175	(22.6)	230	(38.4)	47,072	26	(14.9)	149	(85.1)
Senegal	44	12,034	0^f^	(0.0)	1	(2.3)	283	0	(0.0)	0	(0.0)
Somalia	74	8,958	0	(0.0)				0	(0.0)	0	(0.0)
South Sudan	99	9,606	17	(17.2)	40	(48.8)	4,160	0	(0.0)	17	(100)
Sudan	142	32,376	87	(61.3)	39	(70.9)	7,266	23	(26.4)	64	(73.6)
Tanzania	120	43,494	55	(45.8)	45	(69.2)	17,976	3	(5.5)	52	(94.5)
The Gambia	43	1,719	39	(90.7)	0	(0.0)	0	38	(97.4)	1	(0.0)
Togo	30	5,944	28	(93.3)	0	(0.0)	0	25	(89.3)	3	(10.7)
Uganda	112	32,415	35	(31.3)	9	(11.7)	2,113	0	(0.0)	35	(100)
Zambia	65	12,004	24	(36.9)	27	(65.9)	3,871	4	(16.7)	20	(83.3)
**Total**	**5,308**	**804,128**	**1,024**	**(19.3)**	**1,027**	**(24.0)**	**156,915**	**195**	**(20.1)**	**829**	**(88.6)**

a Proportion of unsurveyed districts that are suspected endemic.

b Proportion of known endemic districts falling into each category of endemicity.

c Unit of implementation (health district) is defined as the first administrative level.

d Third administrative level (wereda) is the implementation unit, but some zonal data are included in this table and used to inform SAFE implementation.

e Five districts were historically endemic in Morocco.

f Regional data available in Table 6.

g TT estimates are in the whole population (0–99 years).

h Data in Egypt were collected at the governorate (regional) level, there have been no recent surveys at finer spatial scales and no alternative public health districts have been defined.

**Table 5:** Availability of current region level trachomatous inflammation–follicular (TF) data from population based prevalence surveys (PBPS) in Africa in children aged 1–9 years.

						Prevalence of TF from PBPS											
			Regions with current regional-level PBPS			<5%			5–9.9%			10–29.9%			>30%		
Country	Total number regions	Total pop (000s)	Regions		Pop (000s)	Regions		Pop (000s)	Regions		Pop (000s)	Regions		Pop (000s)	Regions		Pop (000s)
			n	(%)		n	(%)		n	(%)		n	(%)		n	(%)	
Ethiopia	11	86,132	11	(100)	82,835	5	(45.5)	6,400	0	(0.0)	0	5	(45.5)	55,192	1	(9.0)	21,243
Mozambique^a^	11	22,467	3	(27.3)	4,435	0	(0.0)	0	0	(0.0)	0	2	(66.7)	3,141	1	(33.3)	1,294
Senegal	14	12,034	10	(71.4)	11,107	3	(30.0)	3,560	4	(40.0)	3,632	3	(30.0)	3,916	0	(0.0)	0

a Two surveys in Mozambique were conducted in “super” districts consisting of larger, aggregated geographical areas. An average value has been used for this analysis.

**Table 6:** Availability of current region level trachomatous trichiasis (TT) data from population based prevalence surveys (PBPS) in Africa in adults aged greater than 15 years.

						Prevalence of TT from PBPS			
			Regions with current regional-level PBPS			<0.1%		≥0.1%	
Country	Total number regions	Total pop (000s)	Regions		Pop (000s)	Regions		Regions	
			n	%		n	(%)	n	(%)
Senegal	14	12,034	9	(64.3)	11,108	0	(0.0)	9	(100)

### Distribution of trachoma and population at risk

**Figure 3:**
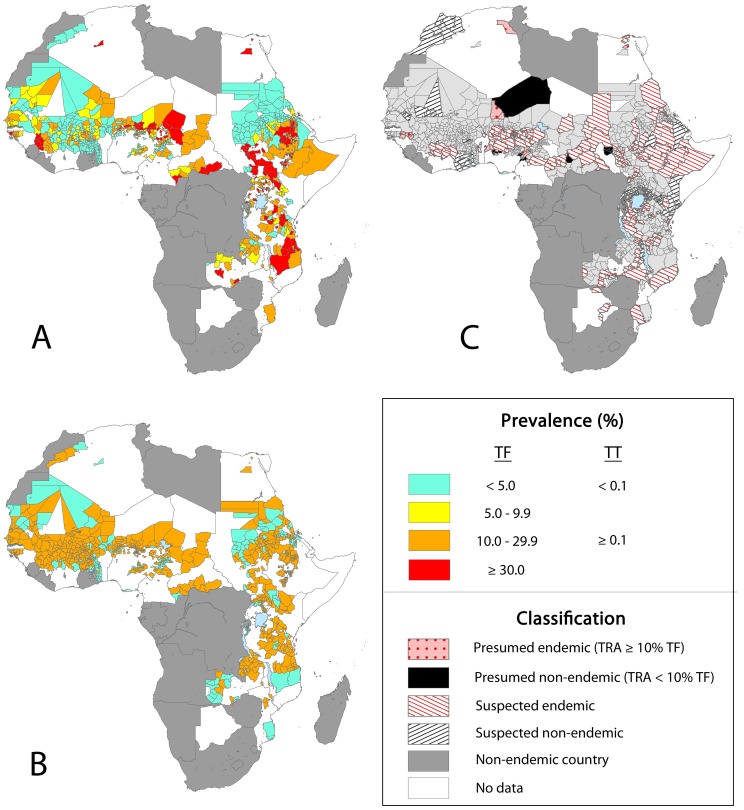
Empirical prevalence of A) trachomatous inflammation–follicular (TF) and B) trachomatous trichiasis (TT) and C) areas of suspected and presumed endemicity in Africa between 1985–2012. Population based prevalence surveys generated data for 1095 districts and 24 regions, while TRA surveys provided information on endemicity for 101 additional districts.

The geographical distribution of trachoma in Africa varies between regions. Trachoma is believed to be endemic in 33 of the 56 countries in Africa, which are mainly located in east and west sub-Saharan Africa, north Africa and a few endemic coastal countries in central Africa (Figure 3).

Based on available data, the highest prevalence of active trachoma and trichiasis remains in the Sahel area of west Africa and Savannah areas of east and central Africa (Tables 3–6). A high proportion of surveyed districts are hyperendemic (defined as TF prevalence in 1–9 year-olds of ≥30%) in South Sudan (83%), Ethiopia (64%), Guinea (50%), Uganda (37%), Chad (38%), CAR (38%) and Tanzania (32%), but large areas suspected to be endemic remain unmapped in each of these countries. West African countries have been the focus of a number of national surveys in the last decade (Figure 1) providing both pre- and post-intervention data for a high proportion of districts in Burkina Faso, The Gambia, Ghana, Mali and Mauritania. Many countries in Central Africa continue to lack data, making estimation of the burden in this region difficult. Based on survey data currently included in the atlas and population estimates, an estimated 129.4 million people live in areas that are confirmed empirically to be trachoma endemic (based on district-level prevalence of TF in 1–9 year-olds greater than 5%) and a further 155 million in areas suspected to be endemic (Table 3). The latter is likely to be a conservative estimate, as it only includes areas classed as suspected endemic based on available TRA or anecdotal information about cases presenting to the health care system. A substantial burden of disease is likely in Chad, Ethiopia and Nigeria due to their large populations in areas of high endemicity (Table 3).

As a direct consequence of repeated infections, the burden of TT follows similar geographical trends to TF within Africa. However, there is a significant backlog of TT surgeries remaining in countries with historically high endemicity levels (Tables 4 & 6). These countries include Ghana and Morocco which, despite success in reducing the burden of active disease, continue to have a high burden of TT arising from both prevalent and incident cases.

### Future mapping needs

Nearly a sixth (13.7%) of surveyed districts fall in the 5–10% TF prevalence category which indicates that they may require higher resolution mapping at the subdistrict level to target MDA to disease foci. In many countries, particularly Central African Republic, Ethiopia, South Sudan, Tanzania and Zambia, there remain a large proportion of unmapped districts that are suspected to be endemic based on higher level prevalence surveys, health systems data or rapid assessments. Based on median TF prevalence in 1–9 year-olds of >20% in surveyed districts (where more time may be needed for control activities to reduce disease prevalence to below elimination thresholds), countries which should be prioritized to finish mapping include Chad, Egypt (based on limited and outdated data at the regional level), Ethiopia, Guinea, Mozambique, Nigeria, South Sudan, Tanzania, Uganda and Zambia. Several other countries with ongoing control programmes, including Cameroon, Kenya, Malawi and Uganda, have few remaining unmapped districts that are suspected to be endemic for trachoma and mapping could be completed within a shorter time frame (Table 3). The distribution of trichiasis in Africa (Table 4) reflects both the known distribution of trachoma as well as areas where trachoma was historically a public health problem and a backlog of cases remain.

## Discussion

With prevalence estimates for at least parts of 29 of the 33 endemic countries in Africa and for 20.6% of all districts in these countries, the Global Atlas of Trachoma (GAT) represents the most comprehensive resource on the geographical distribution of trachoma and an important planning tool for efforts to finalise the global trachoma map. Based on the current data and population estimates, an estimated 129.4 million people live in areas of Africa that are confirmed to be trachoma endemic (TF prevalence greater than 5% in children) and a further 155 million in areas of Africa suspected to be endemic. This corresponds to 98 million people who live in areas of Africa where the prevalence of active trachoma is known to be greater than 10% and currently require access to the SAFE strategy including annual MDA with azithromycin, and a further 31 million people where treatment may need to be targeted at the subdistrict level (Table 3). Summary data collated for this project are a useful advocacy and planning tool, but change as new data become available and estimates can be refined. Iterations of these data have been used at the global level by the International Task Force for Disease Eradication [21] and the International Coalition for Trachoma Control [22]. The online GAT provides an open access platform for all partners to assess what survey data are already available within Africa and the population at risk of trachoma, but also a means to identify gaps in data where further surveys are required and rapidly assess progress in mapping as these activities are scaled up. This resource assists national programmes in planning interventions and provides visualisation of areas where cross-border transmission could be a concern, as well as providing an effective tool to advocate within a country for additional mapping.

Trachoma endemic countries are concentrated in east and west sub-Saharan Africa, north Africa and a few endemic countries in central sub-Saharan Africa (Figure 3). Variation in risk of trachoma both within and between countries has been linked to socioeconomic factors that are associated with transmission through hygienic behaviours and sanitation, as well as varying climatic conditions [23]–[26]. Current data from the GAT confirm that countries with the highest burden of active trachoma and trichiasis remain in the Sahel and Savannah areas of Africa. Well established control programmes in several west and north African countries are likely to have had an impact on the burden of trachoma in the last decade, with successes in control activities documented in Burkina Faso, The Gambia [27], Ghana [28], [29], Mali [30]–[32], Mauritania [33] and Morocco and highlighted by comparison of current and historical maps available on the GAT website (www.trachomaatlas.org). The Gambia, Ghana and Morocco have now reported achievement of trachoma elimination targets and trachoma is believed to be no longer a public health concern in these countries.

Information from this analysis highlights a number of important next steps for defining the burden of trachoma to inform programmatic action. First, a number of countries have both a high prevalence of active trachoma in mapped areas and a large proportion of unmapped districts that are suspected to be endemic. These countries include Central African Republic, Ethiopia, Nigeria, South Sudan and Tanzania. Second, Chad, Guinea, Mozambique, and Uganda are likely to have sizeable areas of high endemicity contributing to the current magnitude of the burden of trachoma in Africa. Generation of baseline data where required, and commencement of interventions in these countries should be accelerated. Third, prioritising countries that have large populations in highly endemic areas, such as Chad, Ethiopia, and Nigeria, will have a greater impact on the overall burden of disease within the programmatic timeframe. Egypt also may be prioritised, based on this rationale, due to the high endemicity of trachoma found in populous areas by earlier regional surveys and a lack of data excluding other geographical areas. Targeting future survey activities to areas which are likely to be highly endemic will allow the initiation of control activities in those areas in which control of trachoma is likely to take the longest. In addition, 13.7% of surveyed districts lie in the 5–10% prevalence category and may require higher resolution mapping at the subdistrict level to target MDA to disease foci (Table 3). Finally, scaling up surgical interventions for TT alongside MDA poises an important challenge in reducing the burden of disease and is increasingly perceived as a limiting factor in meeting UIG targets. There is a substantial backlog of surgeries in countries with historically high endemicity rates. While the incidence of TT will decrease over time along with the number of active infections, the reduction of TT cases is a main goal of control programmes and necessitates scaling up of surgical services in order to meet UIG targets. This presents a number of logistical challenges and demands on human resources; requiring considerable investment in health infrastructure and training in order to identify TT cases and optimise surgical outcomes in order to achieve a sustainable impact.

It should be recognised that data included in GAT vary in quality and methodology, which limit the comparability of the data. The methods used to collect data (sample size, age groups and sampling method) vary and data are collected over a range of years, in which potential socioeconomic changes could introduce further variation. While differences in the age groups surveyed for TT have been adjusted for, older data may not represent current levels of endemicity where mass antibiotic treatments, TT surgery campaigns and secular trends have had an impact on the prevalence of trachoma. Information on treatment and maps of antibiotic and surgical interventions are available on a partner website developed by the International Coalition for Trachoma Control (http://www.trachomacoalition.org/). In practice, these detailed data are assessed contextually and used alongside treatment data to make mapping decisions within a country. Prevalence estimates are rarely reported with confidence intervals, limiting our ability to assess their precision. Generally, precision for TT prevalence estimates is likely to be low as surveys are usually powered only to provide estimates for active trachoma. In addition, the sampling frame of population-based prevalence surveys often excludes urban areas, which are commonly perceived to be at lower risk. These urban populations are typically defined locally and thus vary between countries and districts. Urban populations were included in estimates of the population at risk, due to (i) a lack of reliable evidence that there is no risk of trachoma in urban areas and (ii) the absence of a comparable definition of urban with which to identify these populations. However, in contexts where non-surveyed urban populations have a different risk of trachoma, this decision will result in an under- or over-estimation of the population at risk. The wide prevalence bands used to display these data minimizes the effect of this imprecision and of variation in survey methodologies. Future work could include methods, such as small area estimation, to estimate uncertainty and provide realistic confidence intervals for population estimates [34]. And finally, the estimate of population at risk in areas suspected to be endemic do not include populations of countries currently classified as endemic, but for which no data are currently available (ie Botswana, Somalia and Djibouti).

While much of the available survey data in Africa have helped to inform trachoma control activities, some survey data have not been used to inform control due to limited resources, outdated prevalence data or use of unreliable sampling methodologies. Where prevalence data are felt to be unusable because of their age or the methods used for their collection, the corresponding areas will need to be resurveyed. Variation in the geographical scale at which surveys are conducted introduces a further level of complexity. While the unit of implementation is defined by WHO as the district (which generally corresponds to the second administrative level), in some cases the region (first administrative level) is used instead. Recent recommendations allow data from larger geographic areas (e.g. regions) to justify programme launch in areas where local knowledge or higher level data demonstrate that trachoma is widespread and highly endemic, as was the case in Unity state in South Sudan and Amhara region in Ethiopia [35], [36]. Much historical data in west Africa are representative at regional level and thus not directly comparable to district level data. In addition, it is well established that trachoma is a focal disease and varies with individual and community level risk factors [24], [37]–[39]. Displaying data aggregated at higher administrative levels belies the small scale spatial heterogeneity of clinical disease and inclusion of more densely populated urban areas, which are likely to have lower risk than rural areas, may overestimate the population at risk. Finally, some areas of countries currently regarded as non-endemic may have small pockets of transmission occurring, such as areas in DRC bordering CAR, South Sudan and Zambia.

This work collates all sub-national data available in trachoma endemic countries into a single GIS database in order to summarise current availability of data and the distribution of trachoma. We hope that the maps generated using the GAT and the results presented in this paper will serve as a planning tool and aid efforts to complete the global trachoma map. Prioritising early survey activities to areas where trachoma is suspected to be highly endemic will allow for additional rounds of MDA, implementation of F&E activities and greater time for programmatic impact before 2020. In addition, a number of key research priorities remain to meet GET2020 objectives. First, a standardized mapping methodology should be agreed upon and rolled out in unmapped districts. Ideally this methodology would balance cost and precision in order to appropriately define treatment requirements and provide prevalence estimates for later assessment of programmatic impact. Second, risk mapping using environmental and socioeconomic factors to predict the distribution of trachoma in areas where TRA and clinical data are not available may help identify areas likely to be endemic. Third, this work can be extended to generate estimates of the actual numbers of individuals with TF and TT, by incorporating detailed information on population structure and age-prevalence curves into the analysis, and to other trachoma endemic regions in Asia and South America. Finally, assessing different approaches to trachoma surveillance will be increasingly important as more districts reach this stage of control.
