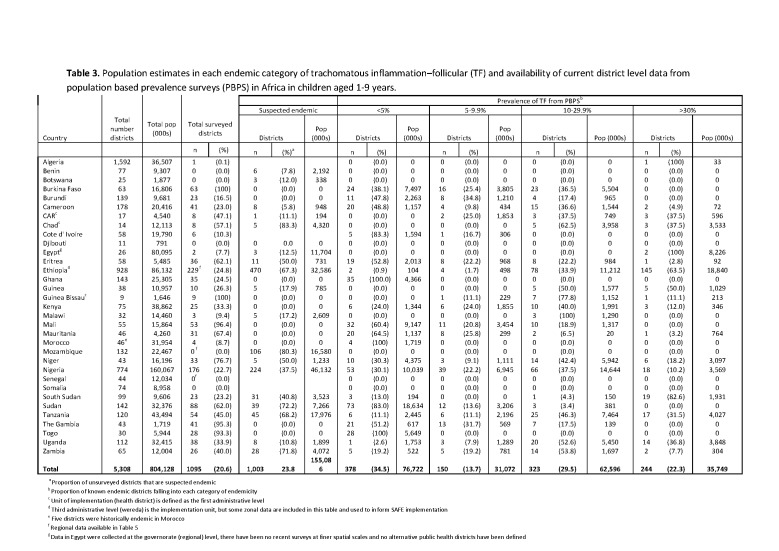# Correction: The Geographical Distribution and Burden of Trachoma in Africa

**DOI:** 10.1371/annotation/f5c644e2-df7e-42ca-9ba4-b4984da715fd

**Published:** 2013-12-13

**Authors:** Jennifer L. Smith, Rebecca M. Flueckiger, Pamela J. Hooper, Sarah Polack, Elizabeth A. Cromwell, Stephanie L. Palmer, Paul M. Emerson, David C. W. Mabey, Anthony W. Solomon, Danny Haddad, Simon J. Brooker

In Table 3 the entry for Ethiopia contains incorrect sums of the subpopulation data within the larger geographical zones. Please see the corrected Table 3 here: 

**Figure:**